# The Benefits of Advanced Botulinum Neurotoxin Injection Training from a Spasticity Management Fellowship

**DOI:** 10.3390/toxins18070301

**Published:** 2026-07-11

**Authors:** John McGuire, Karin Goodfriend, Sarah Golus, Whitney Morelli, Mary Elizabeth S. Nelson-Biersach, Nicholas Ketchum

**Affiliations:** 1 Department of Physical Medicine and Rehabilitation, Medical College of Wisconsin, Milwaukee, WI 53226, USA; kgoodfriend@mcw.edu (K.G.); wmorelli@mcw.edu (W.M.); 2 International Rehabilitation Forum, Middlebury, VT 05753, USA; 3 Center for Neurological Disorders, Greenfield, WI 53228, USA; nicholas.ketchum.md@gmail.com

**Keywords:** spasticity fellowship, spasticity skill acquisition, botulinum neurotoxin (BoNT) injection training

## Abstract

Objective: Residency programs in the United States lack the necessary training for physicians to confidently and competently inject botulinum neurotoxin (BoNT) and manage complex spasticity patients. This creates an unmet need for spasticity patients who could benefit from BoNT injections. This study measured the impact of additional BoNT injection and spasticity management training on post-residency medical practice. Design: Graduates from our Physical Medicine and Rehabilitation residency from 2008 to 2024 were surveyed. Responses were collected from physicians who had residency BoNT injection and spasticity management training (RT) and compared to fellowship BoNT injection and comprehensive spasticity management training (FT). Results: 15 RT and 14 FT physicians completed the survey and were evaluated using descriptive statistics. Spasticity and dystonia treatments comprised an average of 10% of the RT and 62% of the FT clinical practice. BoNT injections involved 5% of the RT practice and 52% of the FT practice. Conclusion: Disabling spasticity is undertreated in this country, partly due to a shortage of training programs for physicians to become skilled and confident at injecting BoNT and comprehensive spasticity management. FT programs can help meet this unmet need by increasing the number of physicians who can provide specialized BoNT injections and spasticity management.

## 1. Introduction

Spasticity affects millions of adults in the United States, and is a significant cause of disability, pain, impaired mobility and reduced quality of life [1,2]. Despite the availability of effective treatment interventions, disabling spasticity remains grossly undertreated [3]. Only 4% of patients who may benefit from botulinum neurotoxin (BoNT) injections receive treatment, and fewer than 10% continued treatment over a two-year period [4,5]. 

These findings suggest a substantial gap between patient need and access to specialized spasticity care.

The American Academy of Physical Medicine and Rehabilitation (AAPMR) released a consensus statement in 2024 highlighting critical gaps in spasticity care; specifically in training, access, and delivery of care [6]. The primary objective of this article focuses on our program addressing the unmet need and expanding the workforce of those who would be proficient at injecting BoNT and managing spasticity.

Most Physical Medicine and Rehabilitation (PM&R) residencies do not have the bandwidth to provide advanced BoNT injection and spasticity management training. As one of the largest spasticity management programs in the country, we have the clinical exposure required for this foundational training. Our program provides dedicated hands-on training for physicians to confidently and competently inject BoNT and manage complex cases of spasticity.

The knowledge and skills needed for physicians to become proficient in comprehensive spasticity management (CSM) are outlined in Table 1. Spasticity is one component of the upper motor neuron syndrome (UMNS) [7]. Understanding the pathophysiology and comprehensive neuromuscular assessment are essential for CSM. Driven by a combination of enhanced excitatory input, diminished inhibitory control and intrinsic changes within the motor neuron pathways, spasticity results from this net hyperexcitability of the stretch reflex [8]. Spasticity, spastic co-contraction, spastic dystonia, synergistic muscle patterns and reflex release phenomena are positive symptoms of UMNS. Negative symptoms of UMNS include muscle weakness, fatigue and impaired motor planning, which may be the major contributor to loss of active function [7]. Rheologic muscle changes also contribute to spasticity and functional impairments.

UMNS presents a complex clinical picture, as spasticity has both advantages and disadvantages [9]. On the beneficial side, it can provide supportive muscle tone, aid in circulatory function and potentially reduce the risk of deep vein thrombosis [10]. However, these benefits are often offset by significant disadvantages, including painful spasms, muscle contractures, skin breakdown, impaired respiratory function and interference with activities of daily living [11]. This dyad emphasizes the importance of individualized assessment and management strategies to optimize function while minimizing complications.

Understanding the origin of spasticity is crucial for selecting the most effective treatment strategies. Tailoring care to the individual and their specific neurological condition ensures a more targeted and successful approach [12]. To design and execute complex treatment plans, clinicians must possess a broad range of skills. Mastery of these essential skills forms the foundation for delivering high quality care to patients with spasticity.

A structured and advanced spasticity training program should highlight the following key domains: nonsurgical intervention, pathophysiology, assessment, optimizing outcomes and goal alignment. Nonsurgical interventions are the cornerstone of CSM. These competencies encompass a range of therapeutic pharmaceutical interventions, beginning with first line treatments [13]. Traditional approaches to spasticity management typically begin with physical and occupational therapy completed with a home exercise program [14]. Oral antispasmodic medications such as baclofen, tizanidine, and dantrolene, are considered first line pharmacologic interventions for focal spasticity [15]. Additionally, gabapentin and pregabalin have shown efficacy in managing spasticity and pain-related symptoms. BoNT injections are widely used for focal spasticity, offering targeted relief with three FDA approved BoNT for spasticity [16]. DNB such as lidocaine and/or bupivacaine can help identify problematic overactive muscles and reveal the benefits of longer acting treatments such as BoNT, phenol and cryoneurolysis [7].

Patients with generalized moderate to severe spasticity can receive significant benefits from intrathecal baclofen (ITB) therapy, particularly those who are unable to tolerate oral baclofen due to side effects [17]. Baclofen is delivered directly to the intrathecal space, allowing for targeted relief with fewer side effects. ITB is often used in combination with BoNT injections and other treatments to enhance overall spasticity management [17].

Emerging as a novel treatment option, cryoneurolysis involves the focal freezing of peripheral nerves to disrupt motor and pain pathways [18]. This technique can lead to reduced spasticity, pain and improved range of motion. Successful application of cryoneurolysis requires advanced training and a high level of proficiency in ultrasound guided procedures, as precise localization of the target nerve is critical to achieving therapeutic efficacy [19].

Neuro-orthopedic surgeries are important to CSM. Common procedures include neurotomy, muscle or tendon lengthening, and transfer procedures which are aimed at reducing spasticity and contractions and improving functional outcomes [20]. While surgical interventions fall outside the scope of PM&R, it is essential for physiatrists to recognize when to involve Neuro-orthopedic colleagues in patient care. Ideally, surgical planning should occur after spasticity has been medically managed [21]. This collaborative approach allows for optimal surgical outcomes and long-term functional improvement [22].

To determine the most appropriate treatment for spasticity, a thorough patient assessment is essential. This highlights the critical role of strong clinical assessment skills in understanding how spasticity affects an individual’s functional abilities. Key tools include standardized spasticity measures such as the Modified Ashworth Scale (MAS) and the Tardieu Scale [23,24]. However, effective evaluation goes beyond these scales. Clinicians must gather a comprehensive view of the patient’s overall condition, including assessments of muscle strength, sensory function, gross and fine motor control, balance, gait, endurance, sleep hygiene, cognitive status and pain levels. This full spectrum assessment ensures that treatment plans are tailored to patient needs and challenges [7].

The final domain focuses on optimizing outcomes while emphasizing clinical judgment, patient-centered communication and interdisciplinary collaboration. This domain involves developing a “keen eye” for identifying the most appropriate treatment options, advocating for patient needs, and aligning care with their individual goals and values. These skills are essential for building trust, fostering interdisciplinary collaboration and delivering patient-centered care in the management of spasticity [17].

Understanding the full spectrum of treatment options is foundational in CSM, but knowing how to deliver those treatments, and in what context, is even more critical [25]. Providing optimal care requires a deep understanding of the individual goals of each patient. Are they seeking improved mobility? Pain reduction? Greater range of motion? Does their orthotic fit properly, or do they need better positioning to assist with dressing?

These are essential questions that must guide clinical decision making. Spasticity management is not a one-size-fits-all specialty; it demands a personalized approach rooted in collaboration, goal-setting and ongoing reassessment [26]. By aligning treatment strategies with the patient’s functional priorities, physicians can deliver care that is both meaningful and effective.

CSM cannot be learned in a one-to-two-month rotation. It is a nuanced specialty that calls for advanced training, critical thinking, and a deep understanding of patient-centered goals and outcomes [27].

To meet the need for advanced spasticity management training, our spasticity fellowship-trained faculty developed a one-month elective spasticity rotation for medical students, a two-month required spasticity rotation for all PM&R residents and a one-year post-residency fellowship. The one-month medical student rotation introduces medical students to the fundamentals of spasticity care. The two-month resident rotation offers additional training for BoNT injections, DNB, ITB and the assessment/management of spasticity. On average, the residents complete 2–3 diagnostic blocks, 4–5 ITB pump refills, 20–30 BoNT injections and 4–6 spasticity consults per week.

After the residents complete their rotation, their skill acquisition and competency are evaluated with a 1–5 entrustment scale looking at BoNT injections and ITB management [28]. This entrustment framework is adapted from the original work of ten Cate and tailored to our program’s specific training objectives [28]. The goal of the resident rotation is to achieve an entrustment level of 3 (act with indirect supervision) on all components of the performance measure, seen in Table 2.

In order to be confident and competent in managing the most complex patients with spasticity, we developed an advanced post-residency spasticity fellowship training program. Fellows engage in diverse clinical settings, including adult and pediatric inpatient and outpatient rotations, sub-specialty clinics, and surgical observation, and can participate in an optional cadaver lab anatomy training. After completion of the fellowship, skill acquisition and fellow competency are evaluated on a five-point entrustment scale (Table 3). The goal is for the fellows to achieve an entrustment level of 4–5 on all components of the performance measure.

Our program incorporates research and education components, encouraging fellows to pursue scholarly projects and contribute to resident didactics. In addition, fellows are supported in attending national PM&R conferences and participating in advanced procedural training, including cryoneurolysis and ultrasound-guided interventions. This comprehensive approach ensures that graduates get more than basic exposure. In this program, fellows get hands on experience, become clinically proficient and procedurally equipped to lead, and advance the practice for the medical uses of BoNT and treatment of spasticity patients.

While some may question the necessity of advanced training in complex spasticity care, it is vital to remember AAPMR’s call to action regarding the lack of physicians competent to provide complex spasticity care [6]. The purpose of this study was to evaluate the impact of a spasticity fellowship training program on physician confidence, procedural exposure and clinical practice involvement in complex spasticity management. We hypothesized that fellowship spasticity-trained (FT) PM&R graduates would demonstrate greater confidence and clinical competence in the management of complex spasticity patients and injection of BoNT compared with residency spasticity-trained (RT) PM&R graduates.

## 2. Results

### 2.1. RT Results

Surveys were distributed to 69 graduates of our PM&R residency program who did not complete a spasticity fellowship. A total of 15 individuals completed the survey, yielding a response rate of approximately 21.7%. RT data is shown in Table 4. Among the respondent residents, spasticity and dystonia treatments comprised an average of 10% of their clinical practice. Only 5% of their practice involved BoNT injections, while none reported involvement in ITB management. When asked to rate their comfort level in managing complex spasticity cases, the average score was 2.6 out of 5, indicating a moderate level of confidence.

Notably, 7 out of the 15 respondents (46.7%) expressed that a dedicated spasticity fellowship would be necessary to adequately prepare them for advanced spasticity management and BoNT injections. Several residents emphasized the value of fellowship training in spasticity management when asked if a spasticity fellowship was necessary. One respondent noted, “Yes, if spasticity management is something you want to be a large part of your practice.” Another added, “Yes, especially if injecting BoNT and ITB pump management is going to be a part of future practice.”

### 2.2. FT Results

The same survey was distributed to 14 of the 16 physicians who completed the comprehensive spasticity management fellowship at our institution. All respondents completed the full set of survey questions. As seen in Table 5, 62% of their clinical practice involves the treatment of spasticity or dystonia, with 53% specifically dedicated to BoNT injections. Additionally, 7% of their practice includes ITB management.

One fellowship-trained physician emphasized the importance of advanced training, stating: “Yes, I think to master BoNT injections and overall optimize spasticity management a fellowship is absolutely necessary. Residency training does not provide enough experience to help you master these skills. Similar to why a pain fellowship is necessary to perfect interventional pain procedure skills, a spasticity fellowship is necessary to perfect interventional skills for spasticity management.”

**Figure 1:**
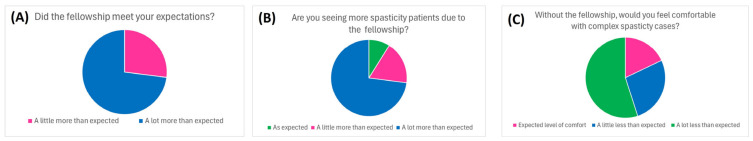
Survey findings from the FT physicians focusing on their expectations (**A**), training outcomes (**B**), and confidence in managing complex spasticity cases (**C**).

**Figure 2:**
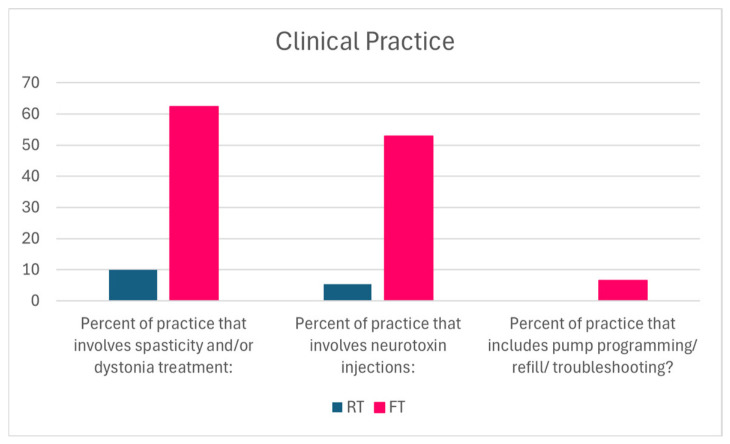
Overview of clinical practice patterns between RT and FT physicians.

**Figure 3:**
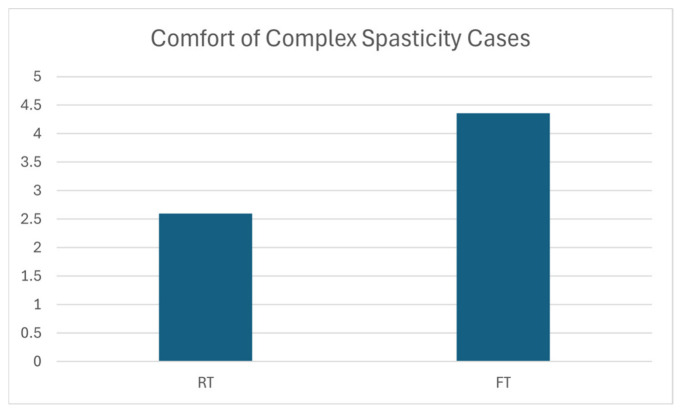
RT compared to FT comfort level with complex spasticity cases.

FT reported an average confidence level of 4.36 out of 5 in managing complex spasticity cases. Of the 14 respondents, 11 (78.6%) indicated that completing a fellowship was necessary to achieve this level of confidence. The remaining three respondents noted that a fellowship may not be essential if spasticity comprises less than 50% of a physician’s practice or if robust training with expert-level mentors was received during residency. Figure 1 illustrates additional FT data. Figure 2 and Figure 3 show a side-by-side comparison of RT and FT survey responses.

## 3. Discussion

To date, spasticity training requirements for PM&R residencies are lacking advanced procedural requirements. Many programs do not have the bandwidth to provide advanced training. Currently, the Accreditation Council for Graduate Medical Education (ACGME) requires that all PM&R residents complete 50 BoNT injections with 10 of those having the potential to be simulated. Additionally, ACGME requires residents to complete five ITB procedures, which can also all be done via simulation, which limits exposure to one of the more advanced treatment options [29]. AAPMR does offer the STEP Interventional Spasticity Certificate Program, which is a specialized training pathway that includes a requirement of 50 BoNT injections, however this is still insufficient for complex spasticity management [30].

It is important to point out structural barriers across PM&R residency programs. Many programs lack access to key modalities such as ITB, phenol procedures, cryoneurolysis or ultrasound guidance. The lack of access to these procedures limits the residents exposure and the ability to get proper training with these modalities. Due to this, we believe that these milestones for residency were set low to accommodate all accredited programs who might not have the procedural volumes. This limited exposure truly does not reflect residents clinical readiness.

To bridge the gap in lack of spasticity management providers, we must move beyond the minimum competency requirements and instead move toward expertise. Basic care may be achieved by any graduate of a PM&R residency, but many patients require complex, expert care. Comprehensive spasticity management in complex patients cannot be learned in a one-month rotation or through electives as not all PM&R residency programs have dedicated spasticity rotations. It is a nuanced specialty that calls for advanced training, critical thinking and a deep understanding of patient-centered outcomes.

With yearlong advanced training, FT physicians reported higher confidence with BoNT dosing and injection strategies, DNB, chemoneurolysis, ITB pump programming and troubleshooting. Their comfort level far exceeded that of residents, reflecting the depth and intensity of fellowship training.

When comparing clinical practice patterns related to spasticity treatment, FT physicians reported significantly greater involvement than RT. On average, FT dedicated 6 times more of their practice to spasticity and dystonia care (62% vs. 10%). Similarly, the proportion of practice involving BoNT injections was 10 times higher among FT (53% vs. 5%). Notably, while 7% of FT practice included ITB management, RT reported no involvement in ITB-related care.

This highlights a significant gap in training and clinical exposure among generally trained physiatrists, reinforcing concerns previously raised by AAPM&R [6]. In this single-institution sample, residents reported that less than 10% of their practice involved spasticity care; mirroring national data indicating that fewer than 10% of patients receive more than two years of injections [4]. Additionally, only 5% of general physiatrists are administering BoNT injections, which closely aligns with the statistic that only 4% of patients receive the BoNT care they need [5]. These findings show the need for expanded training opportunities and specialized fellowship programs to address the clinical demand for BoNT treatments and optimize patient outcomes.

**Figure 4:**
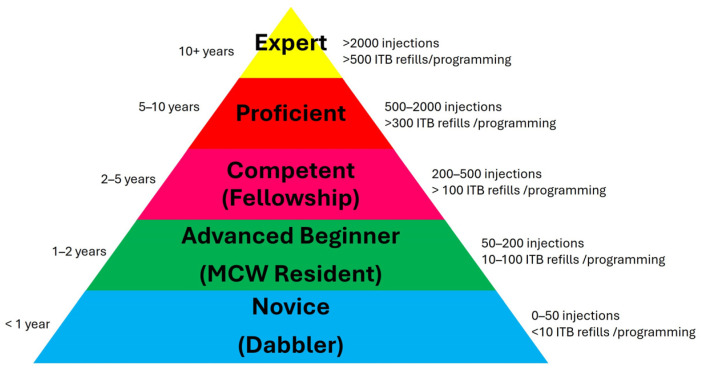
Modified rendition of the Dreyfus Model of Skill Acquisition to reflect BoNT and ITB management.

Our findings support the fact that there is a clear gap in spasticity care delivery, stressing the need for specialized training to achieve expert-level proficiency. FT physicians demonstrate significantly greater involvement and confidence in managing complex cases, particularly with BoNT and ITB procedures. To conceptualize this progression, we propose a modified rendition of the Dreyfus Model of Skill Acquisition, tailored to reflect the stages required to develop expertise in BoNT-based spasticity management (Figure 4) [31]. This framework acknowledges that residency alone may not provide sufficient exposure or procedural depth, and that fellowship training plays a critical role in advancing physicians from novice to expert.

Based on the ACGME guidelines, all PM&R residents are expected to graduate at the novice level in spasticity management [29]. At our institution, a robust two-month spasticity rotation with specific milestones was created. The design of this rotation allows residents to progress to the advanced beginner stage for some aspects of spasticity management, primarily based on the number of BoNT injections performed during training.

Progression beyond residency requires more extensive procedural experience. A dedicated spasticity fellowship typically provides exposure to over 500 BoNT injections, equating to approximately 2–5 years of focused experience, which supports advancement to the competent level. Our belief that physicians with 5–10 years of injecting BoNT in spasticity care are considered proficient, demonstrating consistent performance and clinical judgment across complex cases. Finally, those with 10,000 BoNT injections or more than 10 years of experience reach the expert level, capable of mastery of advanced techniques. This model emphasizes the importance of structured, longitudinal training to achieve true expertise in BoNT-based spasticity management.

To address the current shortfall in complex spasticity care, we must enhance training at the residency level. This would include integrating standardized competencies in spasticity management, BoNT injections, ITB troubleshooting, pump adjustments and refill procedures into the core PM&R curriculum. However, residency alone is insufficient to meet the growing complexity and volume of spasticity care. Currently there are only four PM&R programs with a dedicated spasticity fellowship in the United States. Our fellowship program at the Medical College of Wisconsin was the first to be developed, with the successful training of 17 fellows since 2008. Thomas Jefferson University started in 2018 and has trained 8 fellows. The University of Texas Health Houston and University of Texas Southwestern Medical Center recently added a dedicated spasticity fellowship. Despite the growth of spasticity fellowships, more programs are needed to address the unmet need. With 124 PM&R residency programs nationally, the number of spasticity fellowships should far exceed four [32]. Expanding the number of advanced training programs is essential and these fellowships should be guided by a standardized national curriculum, while allowing for individualized learning pathways based on the physicians background and career goals.

To further formalize this, the development of a board certification in spasticity management is recommended. Such a credential would validate clinical expertise, promote consistency in care delivery and elevate the visibility of spasticity management as a critical domain within PM&R.

This study has several limitations. The small sample size limits statistical power, therefore statistical significance testing was not performed. The broad range of graduation years also introduces variability, as ACGME training requirements may have changed over time, potentially affecting experiences.

## 4. Conclusions

Disabling spasticity remains significantly undertreated in the United States, partly due to a shortage of training programs that prepare physicians with the necessary confidence and competence to inject BoNT and manage complex spasticity patients. The limited number of spasticity fellowships restricts the pipeline of physicians capable of delivering specialized care for CSM, contributing to unmet patient needs and fragmented service delivery.

Expanding the availability of spasticity fellowships would help bridge this gap by increasing the number of physicians who can provide advance BoNT injections and enhance the quality of care for CSM. The establishment of a formal board certification in spasticity management could reinforce clinical standards and add value to the broader field of PM&R.

Future research should explore whether similar disparities in clinical exposure (see more; do more), confidence and practice patterns exist between RT and FT physiatrists across multiple institutions. Such studies could inform national curriculum development and guide strategic expansion of spasticity management and BoNT injection training programs.

## 5. Methods

### 5.1. Research Design

An exploratory survey was sent to participants who (1) graduated from our PM&R residency but who had not completed the spasticity fellowship and (2) those who completed our comprehensive neurorehabilitation and spasticity fellowship. The survey comprised 20 questions, including multiple-choice items, yes/no responses and open-ended prompts. All procedures received institutional approval from the Medical College of Wisconsin (PRO-56086) in accordance with the Declaration of Helsinki. This project had direct contact with subjects and an informational letter was provided to those invited to complete the survey. Participation was voluntary and survey answers were anonymous.

### 5.2. Statistical Analysis

Descriptive analysis was conducted using SPSS Statistics, version 28. Key summary metrics including minimum, maximum and mean values were calculated to characterize the dataset. Open ended questions were analyzed qualitatively using thematic analysis by all authors. This comparative analysis aimed to evaluate the preparedness and clinical confidence between the two groups.

## Figures and Tables

**Table 1:** Overview of skills and knowledge needed for physicians to become proficient in spasticity management.

Domain	Key Components
Nonsurgical Intervention	Oral antispasmodics
	Diagnostic Nerve Blocks (DNB)
	Botulinum neurotoxin (BoNT) injections
	Cryoneurolysis/ Phenol
	Intrathecal baclofen (ITB)
	Physical/occupational therapy
	Home exercise programs
Pathophysiology and Assessment	Pathophysiology
	Comprehensive neuromuscular assessment
	Standardized spasticity measures
	Functional Evaluation
Optimizing Outcomes	Clinical judgment
	Patient-centered communication
	Interdisciplinary collaboration
	Goal alignment
	Outcome measures

**Table 2:** Resident entrustment scale and performance measures.

Entrustment Scale	Resident Performance	Entrustment Goal
1. Be present and observe	BoNT–Upper Limb (minimum 50)	3
2. Act with direct supervision	BoNT–Lower Limb (minimum 50)	3
3. Act with indirect supervision	BoNT–Cervical, other (minimum 50)	3
4. Act without supervision	ITB–Programming (minimum 20)	3
5. Provide supervision	ITB–Refills (minimum 10)	3

**Table 3:** Fellow entrustment scale and performance measures.

	Fellow Performance	Entrustment Goal
	BoNT–Upper Limb (minimum 150)	4
	BoNT–Lower Limb (minimum 150)	4
Entrustment scale	BoNT–Cervical, other (minimum 150)	4
1. Be present and observe	ITB–Programming (minimum 100)	4
2. Act with direct supervision	ITB–Refills (minimum 50)	4
3. Act with indirect supervision	ITB–Troubleshooting, other	4
4. Act without supervision	Peripheral nerve blocks	4
5. Provide supervision	Phenol chemoneurolysis	4
	Cryoneurolysis	4
	Oral spasticity management	5
	Ultrasound guided injections	5

**Table 4:** RT medical practice breakdown.

	Percent of Practice That Involves Spasticity and/or Dystonia Treatment:	Pts/Week with Spasticity and/or Dystonia Treatment:	Percent of Practice That Involves Neurotoxin Injections:	Pts/Week BoNT	Percent of Practice Pump management	Pts/Week Pump Managememnt
Mean:	10	7	5	6	0	0
Min:	0	0	0	0	0	0
Max:	30	25	5	25	0	0

**Table 5:** FT medical practice breakdown.

	Percent of Practice That Involves Spasticity and/or Dystonia Treatment:	Pts/Week with Spasticity and/or Dystonia Treatment:	Percent of Practice That Involves Neurotoxin Injections:	Pts/Week BoNT	Percent of Practice Pump Management	Pts/Week Pump Managememnt
Mean:	62	22	53	20	7	2
Min:	40	10	40	8	0	0
Max:	90	40	90	40	25	8

## Data Availability

The data presented in this study is available on request from the corresponding author (data is not publicly available due to privacy restrictions).
